# Prognostic value of the C-reactive protein to albumin ratio in colorectal cancer: an updated systematic review and meta-analysis

**DOI:** 10.1186/s12957-021-02253-y

**Published:** 2021-05-01

**Authors:** Chun-Kai Liao, Yen-Lin Yu, Yueh-Chen Lin, Yu-Jen Hsu, Yih-Jong Chern, Jy-Ming Chiang, Jeng-Fu You

**Affiliations:** 1 Division of Colon and Rectal Surgery, Department of Surgery, Chang Gung Memorial Hospital, Linkou, No. 5, Fuxing St., Guishan Dist., Taoyuan, 33305 Taiwan; 2 Division of Colon and Rectal Surgery, Department of Surgery, Chang Gung Memorial Hospital, Keelung branch, No. 222, Maijin Rd., Anle Dist., Keelung City, 204 Taiwan; 3 School of Medicine, Chang Gung University, No. 259, Wenhua 1st Road, Guishan Dist., Taoyuan, 33302 Taiwan

**Keywords:** C-reactive protein to albumin ratio, Colorectal cancer, Meta-analysis, Overall survival, Disease-free survival

## Abstract

**Backgrounds:**

The inflammatory biomarker “C-reactive protein to albumin ratio (CAR)” has been reported to significantly correlate to a variety of human cancers. However, there are conflicting results regarding the prognostic value of CAR in colorectal cancer. Previous studies mainly assessed patients in Eastern countries, so their findings may not be applicable to the Western population. Therefore, this updated meta-analysis aimed to investigate the prognostic value of pre-treatment CAR and outcomes of patients with colorectal cancer.

**Methods:**

We conducted a systematic search for eligible literature until October 31, 2020, using PubMed and Embase databases. Studies assessing pre-treatment CAR and outcomes of colorectal cancer were included. Outcome measures included overall survival, disease-free survival, progression-free survival, and clinicopathological features. The pooled hazard ratios (HR) with 95% confidence intervals (CI) were used as effective values.

**Results:**

A total of 15 studies involving 6329 patients were included in this study. The pooled results indicated that a high pre-treatment CAR was associated with poor overall survival (HR 2.028, 95% CI 1.808−2.275, *p* < 0.001) and poor disease-free survival/progression-free survival (HR 1.768, 95% CI 1.321–2.365, *p* < 0.001). Subgroup analysis revealed a constant prognostic value of the pre-treatment CAR despite different study regions, sample size, cancer stage, treatment methods, or the cut-off value used. We also noted a correlation between high pre-treatment CAR and old age, male sex, colon cancer, advanced stage (III/IV), large tumor size, poor differentiation, elevated carcinoembryonic antigen levels, neutrophil-to-lymphocyte ratio, and the modified Glasgow prognostic score.

**Conclusions:**

High pre-treatment CAR was associated with poor overall survival, disease-free survival, and progression-free survival in colorectal cancer. It can serve as a prognostic marker for colorectal cancer in clinical practice.

**Supplementary Information:**

The online version contains supplementary material available at 10.1186/s12957-021-02253-y.

## Introduction

Colorectal cancer (CRC) is the third most common malignancy worldwide. According to global statistics, 1.93 million new cases were diagnosed in 2020 [1]. Despite advances in treatment, including surgical skills, chemotherapy regimens, and the use of biological agents, the rate of death due to CRC remains higher than that due to other cancers, with an estimated 930,000 deaths and CRC was the second-leading cause of cancer-related deaths in 2020 [1]. Because of the heterogeneous nature of CRC, the treatment strategy and outcomes are diverse, even for tumors with the same stage [2]. A 2018 study that assessed the global patterns and trends in CRC demonstrated a diversity in incidence and a mortality rate up to 10-fold worldwide, with distinct gradients across different levels of human development [3]. Determining the prognostic factors for CRC is a critical issue for making treatment plans for CRC and establishing guidance on how intensely the patient should be followed after index treatment.

It is well known that cancer-related inflammation can aid tumor proliferation, angiogenesis, progression, metastasis, and resistance to chemotherapy [4, 5]. Based on this concept, several serum inflammatory markers have been reported as prognostic biomarkers in different cancer types [6]. Either the solitary or combined use of neutrophils, lymphocytes, monocytes, platelets, C-reactive protein (CRP), and albumin has been associated with CRC survival [7–11]. These markers can be further classified into cellular and protein components. CRP is an acute-phase protein regulated by several pro-inflammatory cytokines such as tumor necrosis factor-alpha, interleukin-1, and interleukin-6 [12]. Albumin is recognized as a nutritional status parameter and is associated with chronic inflammation [13, 14]. The utility of these two protein markers has been classically reported as the Glasgow prognostic score (GPS) and modified Glasgow prognostic score (mGPS). GPS and mGPS have been reported as independent prognostic factors for CRC [15, 16]. Recently, a novel inflammation-based marker composed of the CRP-to-albumin ratio (CAR) was proposed to predict the outcome of patients with severe sepsis [17] and served as a prognostic factor for a variety of human malignancies [18]. However, there are conflicting results regarding the prognostic value of CAR in CRC. Ishizuka et al. noted that the overall survival had significantly increased in the low pre-treatment CAR subgroup compared to that of the high pre-treatment CAR subgroup in their study of 627 patients with colorectal cancer who had undergone elective surgery (HR 2.596, *P* < 0.001) [19]. On the contrary, Zhou et al. demonstrated that the dynamic change in CAR pre- and post-surgery was strongly associated with the overall survival in patients with CRC who had undergone surgery; however, if only the pre-operative CAR data was used, it did not significantly affect the overall survival (HR 1.59, 95% CIs 0.86–2.91) [20]. Previous studies analyzing the correlation between pre-treatment CAR and survival in CRC mainly came from East Asia; therefore, evidence about the application of this biomarker in the Western countries is lacking. Thus, we conducted this updated meta-analysis to assess the association between the pre-treatment value of CAR and outcomes in CRC patients by reviewing the findings of all recently published studies. Moreover, we aimed to identify the clinicopathological features associated with a high CAR.

## Methods

### Search strategy

This study was conducted according to the guidelines of the Preferred Reporting Items for Systematic Reviews and Meta-analysis [21]. A comprehensive search was conducted using PubMed and Embase databases from the earliest records to October 2020. The search terms were as follows: (C-reactive protein to albumin ratio OR C-reactive protein/albumin ratio OR C-reactive protein albumin ratio OR CRP/albumin ratio) AND (colorectal cancer OR rectal cancer OR colon cancer) AND (survival OR outcome OR mortality). The bibliographies of the included trials and related review articles were manually reviewed for potential missing studies. The protocol for this systematic review was registered on INPLASY (https://inplasy.com/), and the registration number is INPLASY202140103. The protocol is available in full on the inplasy.com (10.37766/inplasy2021.4.0103).

### Inclusion and exclusion criteria

The inclusion criteria were as follows: (1) either retrospective or prospective studies reporting the association of pre-treatment CAR and the outcome of CRC patients, (2) participants having pathologically confirmed CRC, (3) CAR data before treatment, (4) include survival outcomes such as overall survival (OS) and/or disease-free survival (DFS)/progression-free survival (PFS) for analysis, (5) provide sufficient information for the extraction of the hazard ratio (HR) and associated 95% confidence intervals (CIs), and (6) studies published in English or Chinese. The exclusion criteria were as follows: (1) lack of sufficient data for extracting HR and the associated 95% CIs; (2) no evaluation of survival outcome; (3) use of post-treatment CAR data; (4) case reports, review articles, or conference abstracts; and (5) studies published in languages other than English or Chinese.

### Data extraction and quality assessment

Two independent reviewers examined all retrieved articles and extracted the data using a predetermined form. The following information was extracted: the first author’s name, year of publication, country of study, tumor location, sample size, gender, mean age, cancer stage, type of treatment, the cut-off value of CAR, outcome measures, mean follow-up times, confounding factors of CAR, and analyzed models. Two reviewers evaluated the methodological quality of the enrolled studies independently, according to the Newcastle–Ottawa Quality Assessment Scale (NOS). The NOS contains nine items in three categories: participant selection (four items), comparability (two items), and exposure (three items). A study can be scored a maximum of 1 point for items in the selection and exposure domains and 2 points for the comparability domain [22]. A study with NOS scores of 7 or higher was defined as a high-quality study. Any discrepancies between the two reviewers were resolved through discussion.

### Statistical analysis

All statistical analyses were performed using Comprehensive Meta-Analysis software, version 3 (Biostat, Englewood, NJ, USA). The HRs and associated 95% CIs were directly extracted from the study while reporting survival analysis of OS or PFS/DFS and pooling the prognostic value of high pre-treatment CAR. The clinicopathological features associated with high CAR were also extracted from the studies if a study reported a correlation between CAR and clinicopathological factors. The heterogeneity between studies was determined using the Cochran *Q*-test and *I*^2^ statistics. *I*^2^ ≥ 50% indicated considerable heterogeneity. A random-effects model was employed to pool the HRs and ORs in this meta-analysis. Funnel plots, Begg’s test, and Egger’s test were used to examine potential publication bias. Statistical significance was defined as *p*-values < 0.05, except for the determination of publication bias, which was employed at *p* < 0.1. To evaluate the influence of publication bias, we used the trim-and-fill method. A sensitivity analysis was performed to confirm the robustness of the pooled results by removing each study individually. Moreover, subgroup analysis was performed to investigate the heterogeneity between eligible studies with the following features: study region (Western or Eastern), sample sizes (< 200 or ≥ 200), stage (stage I–III or stage IV), treatment methods (surgery, chemotherapy, or liver resection only), the cut-off value of CAR (< 0.1 or ≥ 0.1), and study method (multivariate or univariate analysis).

## Results

### Study selection and characteristics

**Fig. 1:**
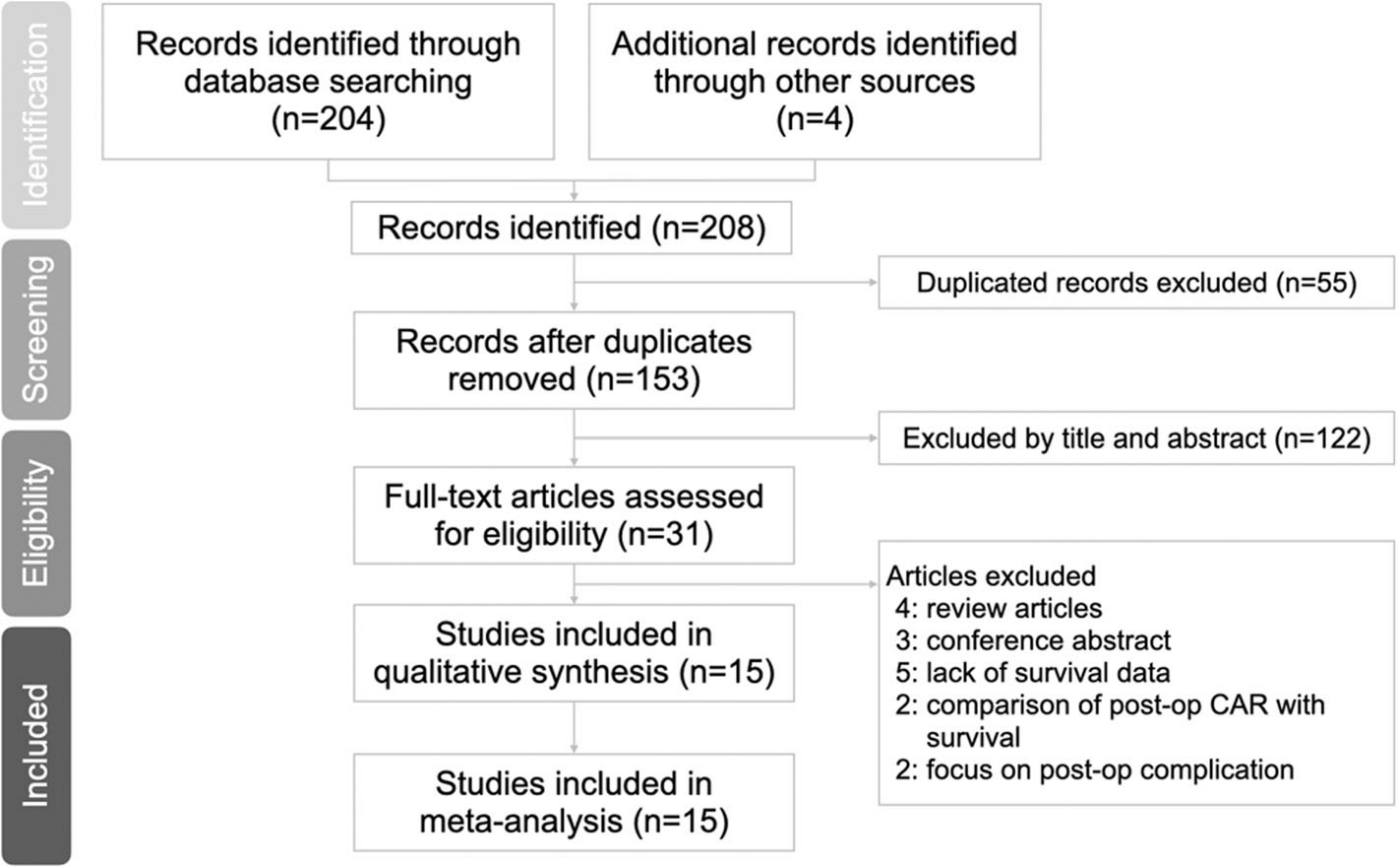
Preferred reporting items for systematic reviews and meta-analyses flow diagram to search and identify included studies

A total of 208 articles were identified based on an online database search and manual search, of which 55 duplicated records were removed. By reviewing the titles and abstracts of the articles, 122 articles were removed. After assessing full-text articles, 16 articles were excluded owing to lack of data to measure survival. Finally, 15 eligible articles were included in the meta-analysis. A flow diagram is shown in Fig. 1.

All included studies were published between 2016 and 2020. The sample sizes of the included studies ranged from 40 to 1303, with 6329 as the total number of enrolled patients. Of these studies, nine were from Japan [19, 23–30], three were from Britain [31–33], two were from China [20, 34], and one was from Korea [35]. The cut-off value of pre-treatment CAR ranged from 0.0278 to 0.6712. There were diverse treatment methods, including surgical resection, chemotherapy regimens, and multidisciplinary treatment. Nine studies consisted of mixed diseases, while five studies consisted only of metastatic disease. The HRs and their 95% CIs were directly extracted from studies on OS and DFS/PFS. The detailed characteristics of the eligible studies are summarized in Table 1 (the confounding factors of CAR are summarized in Additional Table S1).

**Table 1:** Summary of the retrieved studies investigating pre-treatment C-reactive protein to albumin ratio on survival in colorectal cancer patients

Author	Year	Country	Tumor Location	Sample size	Gender, M:F	Age (years)	Stage	Treatment	Cut-off value	Outcome	HR	95% CI	Follow-up time	Analysis	NOS score
Ishizuka	2016	Japan	CRC	627	400:227	NA	0–IV	Surgery	0.038	OS	2.596	1.603–4.204	NA	MV	8
Shibutani	2016	Japan	CRC	99	57:42	63 (27–86)	IV	Chemotherapy	0.183	OS	1.866	1.057–3.295	2.6–73.2	MV	8
Shibutani	2016	Japan	CRC	705	411:294	68 (26-90)	I–III	Surgery	0.0278	DFS CSS	1.503 1.672	1.054–2.143 1.012–2.764	NA	MV	8
Solaini	2016	UK	CRLM	194	125:69	66 (59-73)	IV	Liver resection	0.133	OS	2.04	1.26–3.3	NA	UV	7
Haruki	2017	Japan	CRLM	106	76:30	64.5 (39-87)	IV	Liver resection	0.04	OS DFS	2.559 1.731	1.420–4.613 1.087–2.758	NA	MV	8
Ide	2017	Japan	Rectum	115	82:33	64 (33–83)	I–III	Surgery	0.049	OS DFS	5.09 4.98	2.31–11.58 2.34–11.14	65 (2–189)	MV	8
Dolan	2018	UK	Colon	801	430:371	NA	I–III	Surgery	0.22	OS CSS	1.84 1.76	1.49–2.26 1.31–2.35	NA	MV	7
Climent	2019	Ireland	CRC	566	260:306	69.9 ± 12.3	I–III	Surgery	0.46	OS DFS	1.84 1.73	1.1–3.1 0.8–3.3	NA	UV	6
Shibutani	2019	Japan	CRC	40	25:15	NA	IV	Chemotherapy	0.122	OS PFS	6.478 4.525	1.374–30.537 0.826–24.794	NA	MV	8
Sakamoto	2020	Japan	CRLM	184	121:63	63.1 (25-94)	IV	Liver resection	0.093	OS PFS	2.82 1.62	1.63–4.72 1.02–2.49	NA	MV	8
Son	2020	Korea	CRC	789	486:283	NA	I–III	Surgery	0.14	OS	1.97	1.47–2.64	91 (67–115)	UV	7
Suzuki	2020	Japan	Colon	1303	689:614	65 (26-93)	II–III	Surgery	0.02558	OS DFS	1.8 1.28	1.24–2.68 0.99–1.66	60.2 (3–106)	MV	8
Ni	2016	china	CRC	148	97:51	NA	IV	Chemotherapy	0.6712	OS	2.243	1.450–3.470	NA	MV	8
Zhou	2018	china	CRC	516	331:185	NA	I–IV	Surgery	0.07/0.08	OS DFS	1.59 1.05	0.86–2.91 0.71–1.56	21.72 (2.11–118.72)	MV	8
Tominaga	2016	Japan	Colon	136	79:57	NA	III	Chemotherapy	0.1	DFS	4.43	1.94–10.15	NA	UV	7

*CRC* colorectal cancer, *CRLM* colorectal cancer liver metastasis, *MV* multivariate, *UV* univariate, *OS* overall survival, *DRS* disease-free survival, *CSS* cancer-specific survival, *PFS* progression-free survival

### Quality analysis

The quality of the eligible articles was evaluated by NOS. As shown in additional Table S2, the NOS scores of the included studies ranged from 7 to 8 and were regarded as high quality.

### Meta-analysis of the effect of CAR on overall survival

**Fig. 2:**
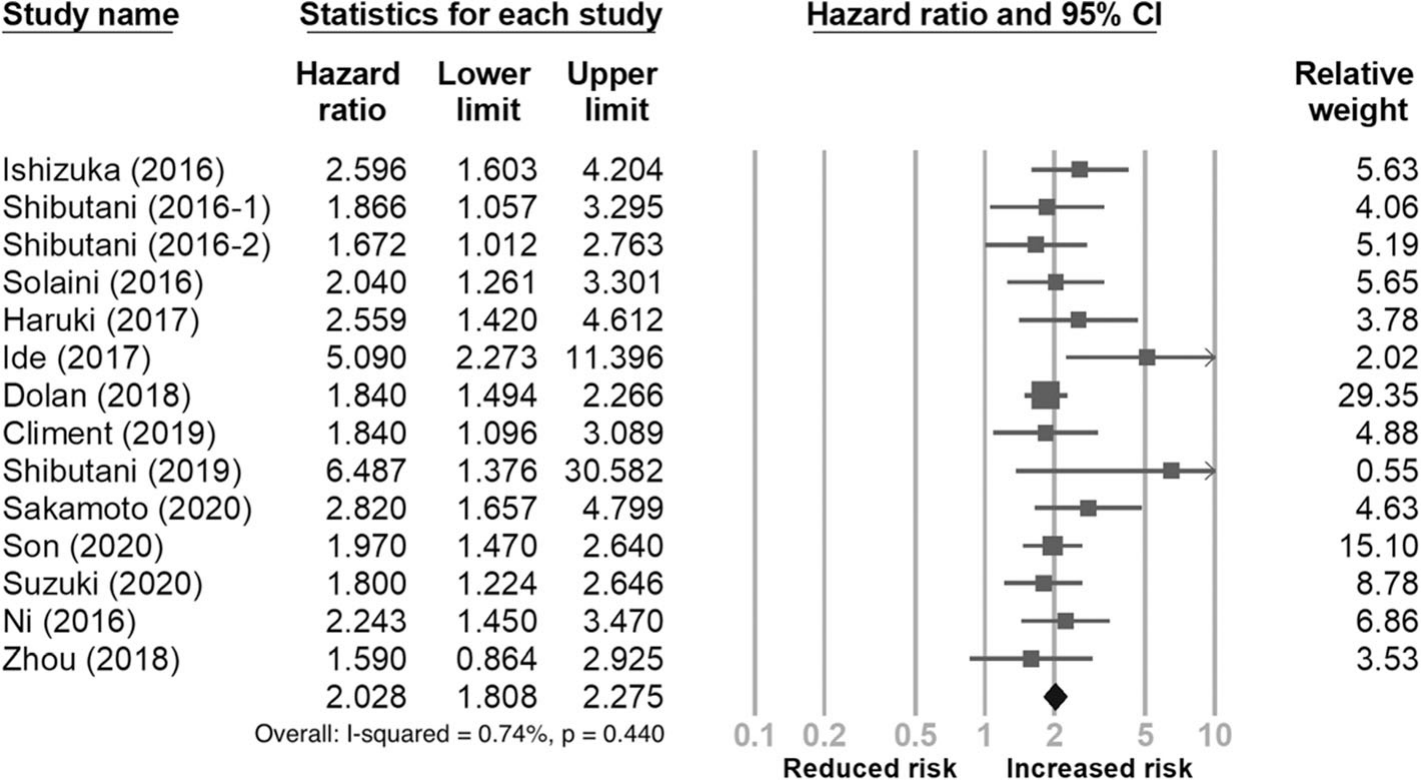
Forest plot of the correlation between the C-reactive protein to albumin ratio and overall survival in patients with colorectal cancer

Fourteen studies [18, 19, 23–29, 31–35] with a total of 6193 participants reported the association of pre-treatment CAR with overall survival. As shown in Fig. 2, the meta-analysis indicated that a high pre-treatment CAR was associated with poor OS (HR 2.028, 95% CI 1.808−2.275). For further evaluation of the prognostic value of CAR on OS, we conducted subgroup analysis using predefined features, and the results are summarized in Table 2. A relatively more significant prognostic effect on OS was observed in studies from the Eastern region, with sample size < 200, stage IV CRC, chemotherapy or liver resection, or using a cut-off value of CAR < 0.1.

**Table 2:** Subgroup analysis of overall survival

Subgroups	No. of studies	Pooled HR (95% CI)	Heterogeneity	
			*I*^2^ (%)	*p* value
Region				
Eastern	11	2.164 (1.838–2.549)	13.78	0.313
Western	3	1.867 (1.560–2.33)	0	0.927
Sample sizes				
< 200	7	2.473 (1.964–3.115)	8.535	0.363
≥ 200	7	1.885 (1.649–2.154)	0	0.878
Stage				
I–III	6	1.926 (1.627–2.281)	19.71	0.285
Include IV	8	2.273 (1.872–2.761)	0	0.682
Treatment				
Surgery	8	1.952 (1.680–2.268)	13.5	0.325
Chemotherapy	3	2.223 (1.539–3.210)	8.89	0.333
Liver resection	3	2.412 (1.778–3.273)	0	0.658
Cut-off value				
≥ 0.1	8	1.924 (1.682–2.200)	0	0.842
< 0.1	6	2.382 (1.812–3.129)	34.427	0.178
Analysis method				
MV	11	2.122 (1.798–2.503)	22.476	0.229
UV	3	1.960 (1.564–2.455)	0	0.958

*CI* confidence interval, *HR* hazard ratio, *MV* multivariate, *UV* univariate

### Sensitivity analysis of CAR on overall survival and publication bias

**Fig. 3:**
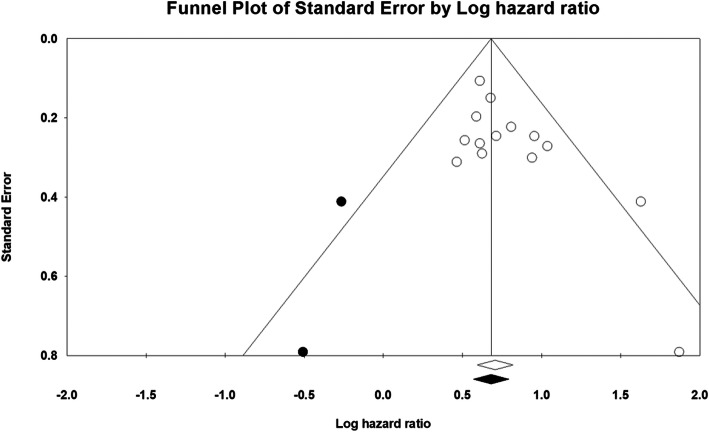
Filled funnel plots for publication bias test of overall survival, the open circles are the real studies; the closed circles are the “filled” studies; the open diamond is the mean effect size of the real studies; the closed diamond is the mean effect size adding the “filled” studies

Sensitivity analysis revealed no substantial impact on the pooled results after removing one study (Additional Figure S1). Publication bias potentially existed, as Begg’s test result was 0.080 and Egger’s test result was 0.022. There were two missing studies in the funnel plot using the trim-and-fill analysis. However, no significant alteration in the pooled result was found after inputting these two studies (HR 2.001, 95% CI 1.733−2.322). The funnel plot is shown in Fig. 3.

### Meta-analysis of the effect of CAR on disease- and progression-free survival

**Fig. 4:**
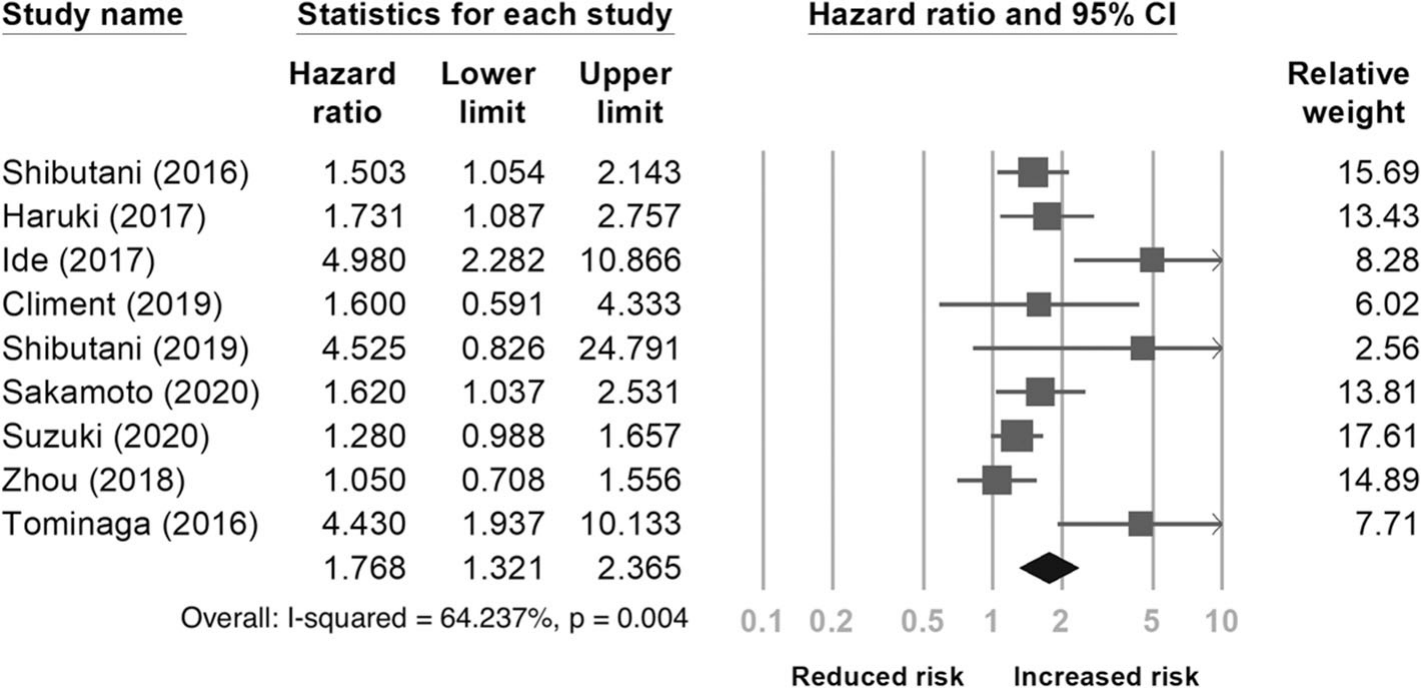
Forest plot of the correlation between the C-reactive protein to albumin ratio and disease-free survival/progression-free survival in patients with colorectal cancer

**Fig. 5:**
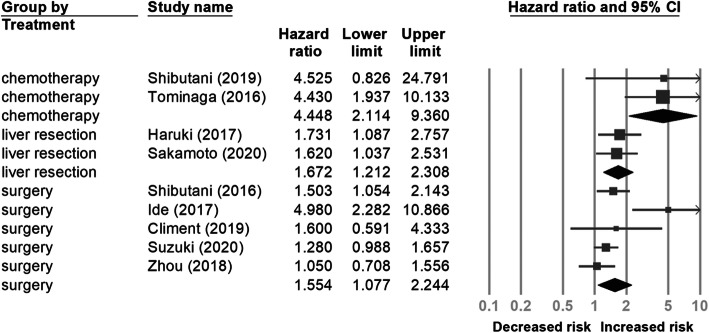
Forest plot for subgroup analysis of the correlation between the C-reactive protein to albumin ratio and disease-free survival/progression-free survival in patients with colorectal cancer according to treatment type

Nine studies [20, 24–30, 33] with a total of 3671 patients provided data on pre-treatment CAR and DFS/PFS. As shown in Fig. 4, the pooled HR showed that a high pre-treatment CAR was associated with poor DFS/PFS (HR 1.768, 95% CI 1.321−2.365). In the subgroup analysis (Table 3), a significant association between high pre-treatment CAR and worse DFS/PFS was observed in the chemotherapy subgroup (HR 4.448, 95% CI 2.114−9.360) (Fig. 5).

**Table 3:** Subgroup analysis of disease-free survival/progression-free survival

Subgroups	No. of studies	Pooled HR (95% CI)	Heterogeneity	
			*I*^2^ (%)	*p* value
Region				
Eastern	8	1.793 (1.314–2.447)	68.69	0.002
Western	1	1.600 (0.591–4.333)	0	1
Sample sizes				
< 200	5	2.646 (1.599–4.378)	61.822	0.033
≥ 200	4	1.289 (1.075–1.546)	0	0.584
Stage				
I–III	5	2.115 (1.297–3.449)	76.339	0.002
Include IV	4	1.477 (1.044–2.089)	40.865	0.167
Treatment				
Surgery	5	1.554 (1.077–2.244)	68.94	0.012
Chemotherapy	2	4.448 (2.114–9.360)	0	0.982
Liver resection	2	1.672 (1.212–2.308)	0	0.84
Cut-off value				
≥ 0.1	4	2.269 (1.215–4.240)	55.379	0.081
< 0.1	5	1.613 (1.129–2.304)	71.106	0.008
Analysis method				
MV	7	1.610 (2.214–2.135)	61.628	0.016
UV	2	2.769 (1.024–7.490)	57.907	0.123

*CI* confidence interval, *HR* hazard ratio, *MV* multivariate, *UV* univariate

### Sensitivity analysis of the effect of CAR on disease- and progression-free survival

The sensitivity analysis also revealed no significant influence on the pooled results after omitting one study (Additional Figure S2).

### Clinicopathological characteristics associated with a high CAR

A total of 11 variables were surveyed in this meta-analysis to determine the association between clinicopathological characteristics and high pre-treatment CAR. Variables included age, sex, tumor location, tumor sidedness, pathological differentiation, stage, tumor size, lymphatic invasion, pre-treatment CEA, mGPS, and NLR. The details of the clinicopathological factors associated with a high pre-treatment CAR are summarized in Additional Table S3. The results revealed that a high pre-treatment CAR was associated with older age (odds ratio [OR] 1.470, *p* = 0.007), male sex (OR 1.452, *p* = 0.001), location (colon vs. rectum, OR 1.724, *p* = 0.008), differentiation (poor vs. moderate/high, OR 1.611, *p* = 0.002), stage (III/IV vs. I/II, OR 2.255, *p* < 0.001), high CEA (OR 2.111, *p* = 0.042), tumor size (> 50 mm vs. < 50 mm, OR 3.687, *p* < 0.001), high NLR (OR 2.452, *p* = 0.002), and advanced mGPS (1 vs. 0, OR 21.405, *p* < 0.001). Conversely, no association was found between high pre-treatment CAR and tumor sidedness or lymphatic invasion. The details of the relationship between high pre-treatment CAR and clinicopathological characteristics are summarized in Table 4.

**Table 4:** Meta-analysis of the association between the C-reactive protein to albumin ratio and clinicopathological characteristics

Characteristic	No. of studies	OR (95% CI)	*p* value	Heterogeneity	
				*I*^2^ (%)	*p* value
Age (> median vs. < median)	4	1.470 (1.028–2.102)	0.007	15.853	0.312
Gender (male vs. female)	7	1.452 (1.167–1.807)	0.001	18.219	0.291
Location (colon vs. rectum)	5	1.724 (1.156–2.570)	0.008	35.14	0.187
Location (right vs. left)	3	1.106 (0.586–2.089)	0.756	49.317	0.139
Differentiation (poor vs. mod./well)	5	1.611 (1.195–2.172)	0.002	0	0.587
Stage (III/IV vs. I/II)	2	2.255 (1.642–3.095)	< 0.001	0	0.688
pre-treatment CEA (high vs. low)^a^	3	2.111 (1.028–4.337)	0.042	85.982	0.001
Tumor size (≥ 50 mm vs. < 50 mm)	1	3.687 (2.608–5.211)	< 0.001	-	-
Lymphatic invasion (yes vs. no)	4	1.020 (0.659–1.579)	0.929	79.615	0.002
NLR (high vs. low)^b^	2	2.452 (1.381–4.354)	0.002	42.753	0.186
mGPS (1 vs. 0)	5	21.405 (6.468–70.835)	< 0.001	66.499	0.018

*CEA* carcinoembryonic antigen, *CI* confidence interval, *mGPS* modified Glasgow prognostic score, *NLR* neutrophil-to-lymphocyte ratio, *OR* odds ratio

^a^Two studies used CEA 5 ng/mL as the cut-off value, and one study used CEA 8.7 ng/mL as the cut-off value

^b^The cut-off values were chosen as 3.0 and 2.9, respectively

## Discussion

We conducted this systematic review and meta-analysis with a total of 6329 participants to determine the prognostic value of CAR in CRC patients. This study included the most recent literature from both Eastern and Western countries. Our study showed that high pre-treatment CAR was associated with the poor survival, either overall or disease-free, of CRC patients. A double death risk is observed more in high pre-treatment CAR patients than in those with low pre-treatment CAR. Moreover, up to four-fold of the progression risk is observed in stage IV CRC patients receiving chemotherapy. The subgroup analysis revealed the constant effect of high pre-treatment CAR on survival, including stratification by region, sample size, stage, treatment type, or CAR cut-off value.

Additionally, a high pre-treatment CAR correlated with many advanced clinicopathological features, such as older age, advanced tumor size, stage, elevated CEA and NLR levels, and prominence more in the colon than in the rectum. In addition to these factors, a high level of CA-199 [19] and an increased tumor depth [23] were reported to be correlated with a high pre-treatment CAR. Consequently, CAR can be used as a prognostic biomarker for patients with CRC. Assessing CAR before treatment is essential not only for its prognostic value but also for stratifying high-risk patients.

It is well known that inflammation plays a critical role in tumorigenesis. Malignancies can trigger an intrinsic inflammatory response that affects the tumor cells’ microenvironment in tumor progression and metastasis [36]. Therefore, several inflammation-related markers have been studied for use in cancer treatment. In recent decades, the neutrophil-to-lymphocyte ratio, platelet-to-lymphocyte ratio, GPS, and mGPS have been widely used to predict CRC outcomes [6, 15, 16]. GPS and mGPS are composed of CRP and albumin, both of which may reflect the underlying inflammatory condition and nutritional status. Because only three scores characterize GPS and mGPS, most patients are categorized to have scores 0 or 1, typically considered to have a better prognosis than score 2. However, by using CAR, which has a quantitative nature with a continuous range of values, we can further divide the GPS/mGPS group with CAR. Ishizuka et al. found a superior overall survival in the low CAR subgroup than in the high CAR subgroup for CRC patients with GPS 0 and GPS 1, respectively [19].

In addition to the predictive role of prognosis in patients with CRC, there are several CAR utilities in clinical practice. A multicenter study conducted by Hashimoto et al. found that high pre-operative CAR was an independent predictor of postoperative complications (HR 2.864, *p* = 0.029) in patients aged 85 years or older who underwent primary tumor resection for CRC [37].

Shibutani et al. found that the normalization of CAR at 8 weeks after initiating chemotherapy for stage IV CRC patients tended to have a better OS than the persistently high CAR subgroup [23]. A study conducted by Zhou et al. also found that dynamic changes in inflammatory markers, including CAR, are associated with OS [20]. Moreover, Tominaga et al. investigated 136 patients with stage III CRC receiving adjuvant chemotherapy and found a significant increase in the risk of severe side effects of chemotherapy (HR 7.06, 95% CI 2.51–19.88, *p* < 0.01) in patients with high pre-treatment CAR [30]. In addition to pre-treatment CAR usage, Ge et al. found that high postoperative CAR on POD-3 had a higher positive predictive value for postoperative complications than CRP alone [38]. Another study conducted by Matsuoka et al. found the survival benefit of adjuvant chemotherapy in stage III CRC patients with high postoperative CAR after curative surgery but no superior outcome of adjuvant chemotherapy in the low postoperative CAR subgroup [39].

As an acute-phase protein, CRP is mediated by many pro-inflammatory cytokines, such as IL-1, IL-6, and TNF-α [12]. These cytokines suppress the synthesis of albumin under inflammatory conditions [13, 14]. Thus, as a combination of these two proteins, CAR may reflect the severity of inflammation, which is believed to be correlated with tumor progression. This updated meta-analysis summarized the most recent studies and indicated the prognostic value of pre-treatment CAR for OS and DFS/PFS. Thus, CAR data should be used for both, checking the pre-treatment status of patients with CRC and other cancers, and observing the dynamic changes in CAR values during and post-treatment. More studies involving CAR are warranted to gain a complete understanding of its utility in CRC treatment.

### Limitations of this meta-analysis

This study has some limitations. First, all of the included studies were retrospective in design, the existence of co-morbidities influenced the CRP and albumin values, and selection bias potentially existed. Second, most of the included studies originated in the Eastern region. The pooled results may not be as consistent as they are in the Western region. Third, the CAR cut-off value varied between each study; therefore, we could not determine the most reliable clinical practice value. Fourth, we could only extract HRs and 95% CIs directly for the meta-analysis from each study. The existence of missing data may reduce the overall accuracy of the pooled results. Finally, publication bias did exist in this systemic review and may attributed to the following reasons. We only included the articles in English and Chinese although we did not set any limitation of language during the searching process. Unpublished papers with non-significant results may exist; however, there was no significant alteration in the pooled result after adding two missing studies in the funnel plot using the trim-and-fill analysis. We believe this meta-analysis is still reliable.

## Conclusion

This meta-analysis indicates that elevated pre-treatment CAR is correlated with poor OS and DFS/PFS in patients with CRC. CAR is a reliable biomarker in clinical practice. It is a predictor of prognosis and stratified high-risk patients from the existing classification.

## Supplementary Information

**Additional file 1: Figure S1.** Sensitivity analysis of C-reactive protein to albumin ratio and overall survival in patients with colorectal cancer

**Additional file 2: Figure S2.** Sensitivity analysis of C-reactive protein to albumin ratio and disease-free survival / progression-free survival in patients with colorectal cancer

**Additional file 3: Table S1.** The confounding factors extracted from studies investigating pre-treatment C-reactive protein to albumin ratio on survival in colorectal cancer patients

**Additional file 4: Table S2.** Scores of the eligible studies according to the Newcastle-Ottawa Scale

**Additional file 5: Table S3.** The detail of retrieving clinicao-pathology factors associated with high pre-treatment CAR

## Data Availability

The datasets generated and analyzed during the current study are available from the corresponding author upon reasonable request.

## Abbreviations

CEA: Carcinoembryonic antigen
CAR: C-reactive protein to albumin ratio
CRC: Colorectal cancer
CI: Confidence interval
DFS: Disease-free survival
GPS: Glasgow prognostic score
HR: Hazard ratio
mGPS: Modified Glasgow prognostic score
NLR: Neutrophil-to-lymphocyte ratio
NOS: Newcastle–Ottawa Quality Assessment Scale
OS: Overall survival
PFS: Progression-free survival
PRISMA: Preferred reporting items for systematic reviews and meta-analyses
