# Supplementary figures and images for: Prognostic value of the C-reactive protein to albumin ratio in colorectal cancer: an updated systematic review and meta-analysis
Source: World J Surg Oncol. 2021 May 1;19:139. doi: 10.1186/s12957-021-02253-y (PMC8088626; doi:10.1186/s12957-021-02253-y)

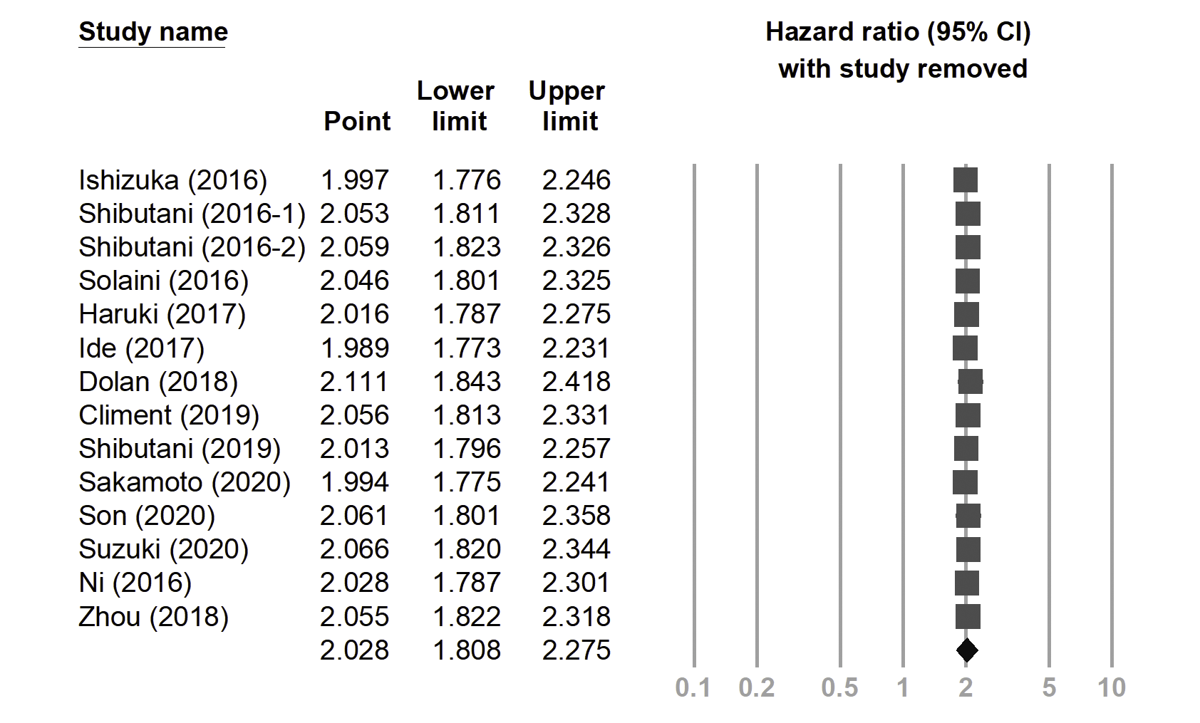

Supplement: Supplementary file 1 — Additional file 1: Figure S1. Sensitivity analysis of C-reactive protein to albumin ratio and overall survival in patients with colorectal cancer [file 12957_2021_2253_MOESM1_ESM.tif]

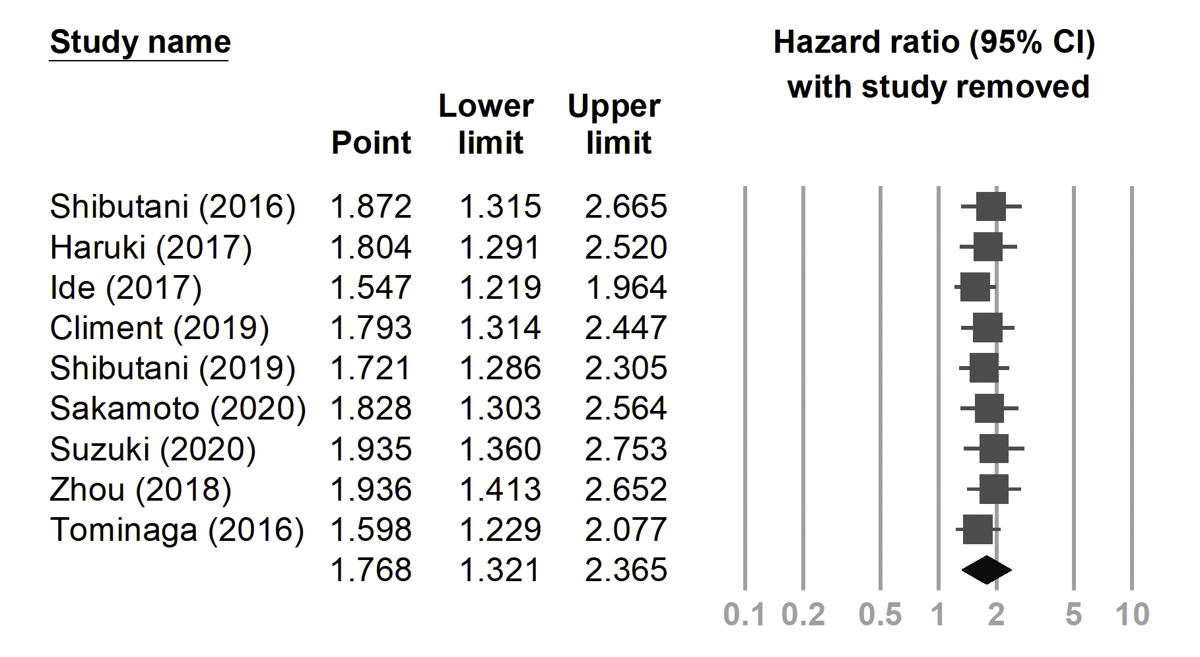

Supplement: Supplementary file 2 — Additional file 2: Figure S2. Sensitivity analysis of C-reactive protein to albumin ratio and disease-free survival / progression-free survival in patients with colorectal cancer [file 12957_2021_2253_MOESM2_ESM.tif]
